# Simulation-based optimisation to quantify heterogeneity of specific ventilation and perfusion in the lung by the Inspired Sinewave Test

**DOI:** 10.1038/s41598-021-92062-w

**Published:** 2021-06-16

**Authors:** M. C. Tran, V. Nguyen, R. Bruce, D. C. Crockett, F. Formenti, P. A. Phan, S. J. Payne, A. D. Farmery

**Affiliations:** 1 Institute of Biomedical Engineering, Department of Engineering Science, University of Oxford, Oxford, OX3 7DQ UK; 2 Nuffield Department of Clinical Neurosciences, John Radcliffe Hospital, Oxford, OX3 9DU UK; 3 Department of Materials and Oxford-Man Institute of Quantitative Finance, University of Oxford, Oxford, OX2 6ED UK; 4 Centre for Human and Applied Physiological Sciences, King’s College London, London, SE1 9RT UK; 5 Department of Biomechanics, University of Nebraska, Omaha, NE USA

**Keywords:** Biomedical engineering, Respiration, Chronic obstructive pulmonary disease, Respiratory distress syndrome, Respiratory tract diseases, Computational models

## Abstract

The degree of specific ventilatory heterogeneity (spatial unevenness of ventilation) of the lung is a useful marker of early structural lung changes which has the potential to detect early-onset disease. The Inspired Sinewave Test (IST) is an established noninvasive ‘gas-distribution’ type of respiratory test capable of measuring the cardiopulmonary parameters. We developed a simulation-based optimisation for the IST, with a simulation of a realistic heterogeneous lung, namely a lognormal distribution of spatial ventilation and perfusion. We tested this method in datasets from 13 anaesthetised pigs (pre and post-lung injury) and 104 human subjects (32 healthy and 72 COPD subjects). The 72 COPD subjects were classified into four COPD phenotypes based on ‘GOLD’ classification. This method allowed IST to identify and quantify heterogeneity of both ventilation and perfusion, permitting diagnostic distinction between health and disease states. In healthy volunteers, we show a linear relationship between the ventilatory heterogeneity versus age (${R}^{2}=0.42$). In a mechanically ventilated pig, IST ventilatory heterogeneity in noninjured and injured lungs was significantly different (*p* < 0.0001). Additionally, measured indices could accurately identify patients with COPD (area under the receiver operating characteristic curve is 0.76, *p* < 0.0001). The IST also could distinguish different phenotypes of COPD with 73% agreement with spirometry.

## Introduction

COPD is the third most frequent cause of death globally^1–3^. In COPD, cellular and structural lung changes develop early in the disease, decades before observable gross airflow obstruction is detected by spirometry^4^. Abnormalities of ventilatory heterogeneity accompany these early structural changes before airflow limitation as measured by spirometry, becomes evident^5^. Considerable benefits can be achieved by early diagnosis and treatment since the most rapid decline in lung function occurs in the early stages^2,6^. Acute lung injury and (ARDS) are typically seen in critically ill patients being treated with mechanical ventilation in Intensive Care Units (ICU). Coronavirus disease 2019 (COVID-19) is an emerging infectious disease that caused a global pandemic^7,8^. In a study of 201 COVID-19 patients, 41.8% of patients developed ARDS and 52.4% of those died^8^. One characteristic of ARDS is significant ventilatory heterogeneity.

Consequently, there is renewed interest in simple bedside/clinic-based measures of ventilatory heterogeneity as a new maker of early lung abnormality and guide for treatment^9,10^. Ventilatory heterogeneity is the unequal distribution of the airflow (ventilation) relative to the size of the lung regions to which it is distributed. This is related to, but distinct from, heterogeneity in regional balances of ventilation relative to perfusion (V/Q heterogeneity)^5^. Several lung tests have been claimed to accurately measure ventilatory heterogeneity^9,11^. These are not yet amenable to widespread use at the bedside or clinic. Of the simple available noninvasive lung function tests, the Multiple Breath Nitrogen Washout technique has been used in the past to investigate the ventilation–volume distribution^11–14^.

The Inspired Sinewave Test (IST) is a non-invasive method to continuously monitor cardiopulmonary indices by using forced oscillations of nitrous oxide (${\mathrm{N}}_{2}\mathrm{O}$) tracer gas signals^15–18^. The IST can measure several lung parameters (E_LV_—effective lung volume, V_D_—deadspace volume and Q_p_—pulmonary blood flow) based on a single, well-mixed compartment model. The ability of IST to detect ventilatory heterogeneity has been assessed in healthy volunteers^19^. This study showed that with increasing heterogeneity, the recovered effective lung volume is reduced with reference to a standard measure (plethysmography). There is potential for further developing the IST to be able to characterise heterogeneity intrinsically, and independently of a second standard measure by using a simulation-based optimisation.

Simulation-based optimisation is a technique used in engineering, science and business research to take advantage of the ability of simulations to model phenomena and systems^20,21^. A simulation could be described as a ‘black-box’ that depends on input–output data from the simulation in its search for optimal input settings. Bayesian optimisation (BO) can speed up the experimental process by using machine learning to guide the search^22,23^.

Therefore, we developed a simulation-based optimisation for the IST to estimate heterogeneity of specific ventilation and perfusion. The method combines the advantages of using a multicompartment lung simulation and Bayesian optimisation to improve performance. We implemented the optimisation in two IST data sets, including human COPD data and experimental animal ARDS data. This is the first study to use IST to evaluate changes in lung heterogeneity in different lung conditions.

## Results

The mean and standard deviation of the model outputs in animal and human data sets are presented in Table 1. There were no significant differences in the *mean* values of the lognormal distributions of either ventilation or perfusion between the normal and abnormal lung in both animal and human studies, i.e. there was no left–right shift in the position of the distributions. However, the standard deviations of the lognormal distributions (${\sigma }_{V}$ and ${\sigma }_{P}$) differ significantly between the normal and the diseased lung, i.e. the distributions widen with disease. Noticeably, the values of ${\sigma }_{V}$ (standard deviation of the ventilation lognormal distribution) shows significant differences in both data sets (*p* < 0.0001 in animal set and *p* < 0.01 in COPD).

**Table 1:** Results of the simulation-based optimisation in three datasets.

Characteristic	Animal			Human		
	Noninjured	Injured	*p*	Healthy	COPD	*p*
$\mathrm{E}\mathrm{L}\mathrm{V}\left(\mathrm{L}\right)$	1.23 (0.54)	0.86 (0.47)	< 0.01	2.3 (0.4)	2.0 (0.5)	< 0.05
${\mathrm{Q}}_{\mathrm{P}}\left(\mathrm{L}/\mathrm{m}\mathrm{i}\mathrm{n}\right)$	2.9 (0.9)	3.2 (1.0)	ns	4.9 (1.8)	4.8 (2.0)	ns
${\mathrm{\mu }}_{\mathrm{V}}$	1.08 (0.23)	1.14 (0.34)	ns	1.14 (0.32)	1.11 (0.36)	ns
${\mathrm{\sigma }}_{\mathrm{V}}$	0.20 (0.04)	0.31 (0.10)	< 0.0001	0.71 (0.16)	0.89 (0.33)	< 0.01
${\mathrm{\mu }}_{\mathrm{P}}$	1.02 (0.33)	1.15 (0.41)	ns	1.21 (0.44)	1.22 (0.31)	ns
${\mathrm{\sigma }}_{\mathrm{p}}$	0.28 (0.11)	0.27 (0.19)	ns	1.01 (0.28)	1.2 (0.28)	< 0.001

Mean and standard deviation are shown. $ELV$: Effective lung volume; ${Q}_{P}$: pulmonary blood flow; ${\mu }_{V}$ and ${\sigma }_{V}$: mean and standard deviation of the ventilation lognormal distribution;${\mu }_{P}$ and ${\sigma }_{P}$: mean and standard deviation of the perfusion lognormal distribution. Student’s *t *test was applied with ns = non-significant.

### Verification of the model in simulated patients

**Figure 1:**
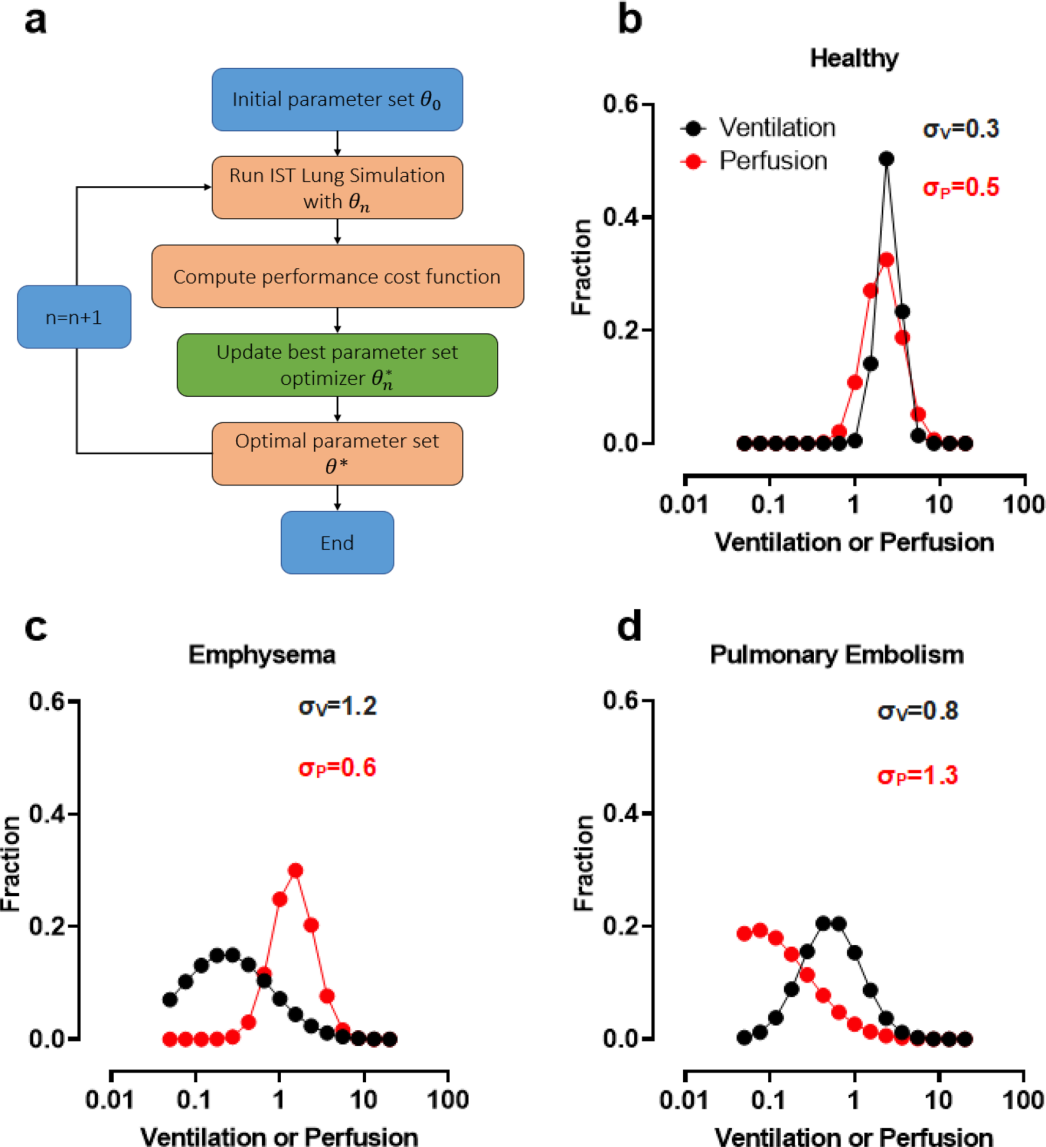
A schematic diagram of the lung simulation-based optimisation and examples of the simulated lognormal distributions. The model incorporated ventilatory and perfusion distributions which were taken from the literature, to represent the healthy individual and patients with emphysema and pulmonary embolism^33^. Panels **b**, **c** and **d** show the recovery of these distributions when a simulated IST is applied to the model.

The lung simulation has been previously developed and verified and was then applied here to complete the simulation-based optimisation model. Firstly, this simulation is developed for the IST. The verification results are included in the appendix. Repeated calculations in the same data were undertaken. Repeated results had a variation of less than $5\%$. A demonstration of the simulated lognormal lung of emphysema and pulmonary embolism are shown in Fig. 1c, d. In Fig. 1c, the emphysema patient has a wider distribution of ventilation (greater heterogeneity) than the healthy one (${\sigma }_{V}=1.2$ compared with ${\sigma }_{V}=0.3$ in healthy). In Fig. 1d, the patient with pulmonary embolism has a wider distribution of both ventilation and perfusion than the healthy case (${\sigma }_{V}=0.8$ and ${\sigma }_{P}=1.3$).

### IST heterogeneity indices in animal data

**Figure 2:**
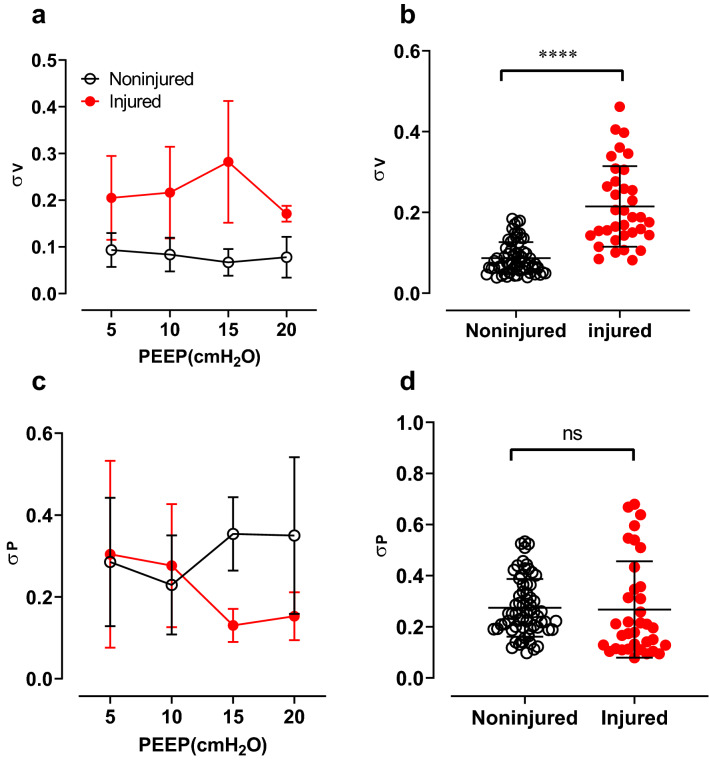
The IST heterogeneity indices in animal data. Panels (**a**) and (**b**) show the distribution of the IST ventilation heterogeneity index (σ_V_) in noninjured and injured lungs. Similarly, panels **c** and **d** show the IST perfusion heterogeneity index (σ_P_). Panels (**a**) and (**c**) present the mean and standard deviation of the IST heterogeneity indices versus PEEP levels. Panels (**b**) and (**d**) present the unpaired Student’s *t *test comparisons in all PEEP levels. ns: non-significant and ****p < 0.0001.

Figure 2 shows the IST heterogeneity results in noninjured versus injured animal data. The measurements were performed at different levels of positive end-expiratory pressure (PEEP). In panel a, the IST ventilation heterogeneity was not significantly influenced by the PEEP level in noninjured animals. Figure 2b shows pooled data separated into noninjured and injured data in all PEEP levels. A significant difference was observed in the IST ventilation heterogeneity (${\sigma }_{V}$), *p* < 0.0001, however, no significance was seen in the IST perfusion heterogeneity (${\sigma }_{P}$) in panel d. However, as shown in panel a and c, although there is no overall significant influence of the PEEP level on lung heterogeneity, at PEEP 15 cmH_2_O, there was a wide separation between noninjured and injured data.

### IST heterogeneity indices in human subjects

**Figure 3:**
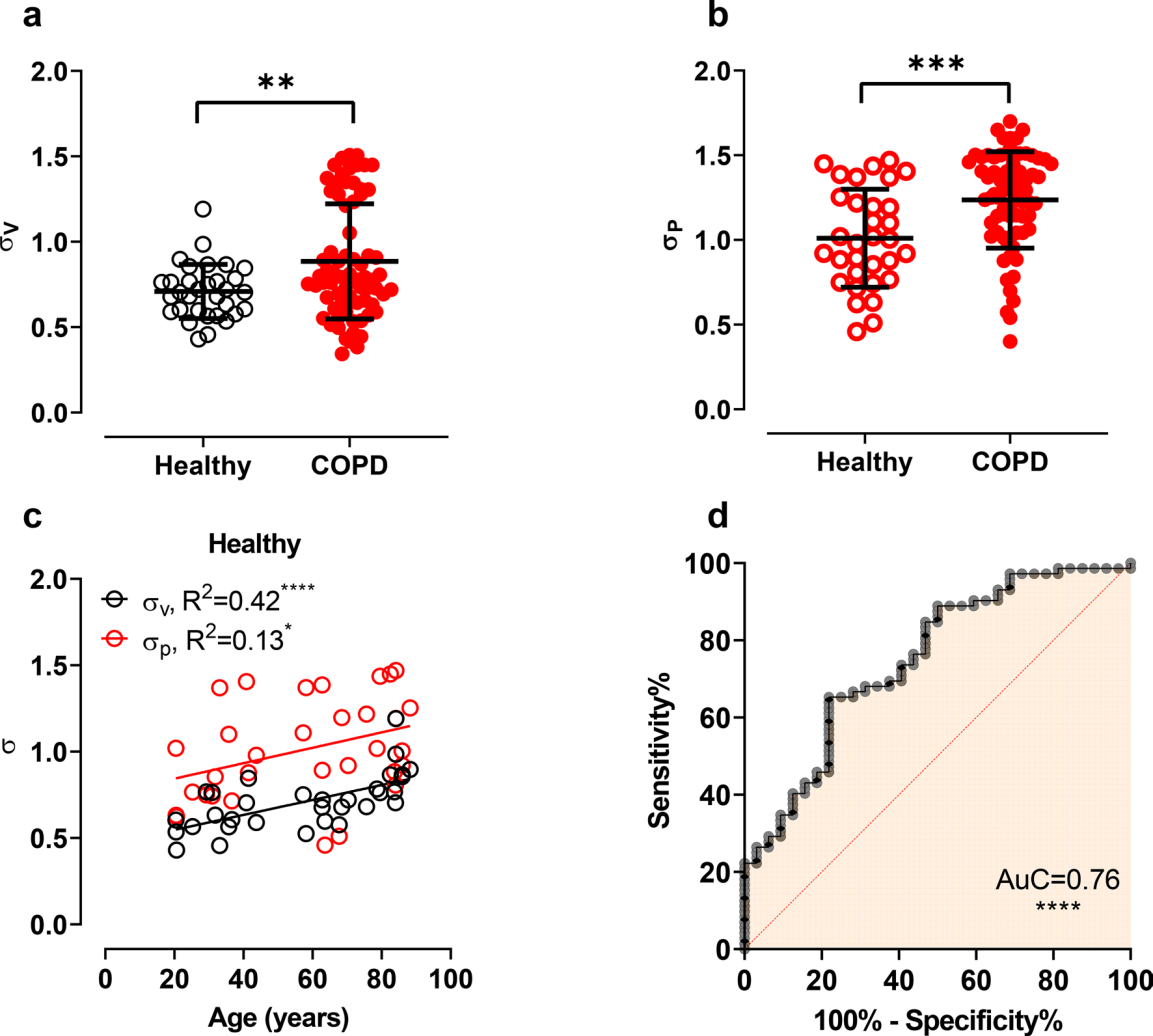
The IST heterogeneity indices in human data. Panels (**a**) and (**b**) show unpaired Student’s *t *test of the IST ventilation (${\sigma }_{V}$) and perfusion heterogeneity (${\sigma }_{P}$) indices. Panel (**c**) shows the linear regression of the IST heterogeneity indices versus ages in healthy people. Panel (**d**) shows the receiver operating characteristic curve of the COPD classification using only IST heterogeneity indices. *p < 0.05; **p < 0.01; ***p < 0.001; ****p < 0.0001.

In the human data set, Fig. 3, the IST heterogeneity indices show the potential to classify healthy versus COPD groups. In panel a and b, results of unpaired t-test illustrate the ability to identify healthy versus COPD subjects. However, the classification was not entirely robust, with an area under the receiver operating characteristic curve of 0.76 (*p* < 0.0001), panel 3d. Interestingly, in Fig. 3a, two distinct sub-populations of COPD phenotype can be observed in the distribution graph of ${\sigma }_{V}$, indicating the wide range of disease severities within the diagnostic label of COPD.

Furthermore, in healthy subjects, Fig. 3c shows the relationship between IST heterogeneity indices and age, which was found to be a linear $\left({R}^{2}=0.42,p<0.0001\right)$. Based on this relationship, we normalised IST heterogeneity to account for the influences of normal ageing in further analysis. The equations are reported below (age in years):

1 $${\sigma }_{V}=0.0043*age+0.46$$

2 $${\sigma }_{P}=0.0045*age+0.75$$

**Figure 4:**
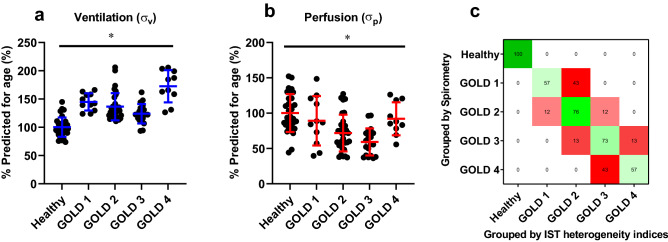
The IST heterogeneity results in the human data set, including healthy and COPD phenotypes (GOLD 1–4). Panels (**a**) and (**b**) show % of measured values over the predicted values for age in different lung conditions. Kruskal–Wallis test was applied for multiple groups comparison with abnormal distribution. Panel (**c**) shows the confusion matrix result of the logistic regression analysis in percentage. This analysis grouped patients by the IST heterogeneity indices and compared them with the classification results using Spirometry (standard method). *p < 0.05.

In Fig. 4, further understanding of the IST lung heterogeneity in different COPD phenotype is revealed. In this figure, % of predicted values for age were calculated according to Eqs. (1) and (2), thereby eliminating the influence of normal ageing. The IST heterogeneity indices (both ventilation and perfusion) of healthy versus COPD patients (*p* < 0.05) are now clearly distinct. However, there was no clear distinction among the COPD groups. Additionally, panel c shows a confusion matrix of patient classification results using only IST heterogeneity indices. The 'GOLD' classification was assumed to present the ground truth. This classification results achieved an accuracy of 73% similarity compared to GOLD classification. The highest predicted accuracy was the healthy group (100% accuracy) and the lowest was COPD phenotype 1 and 4 (57%).

### Bayesian optimisation performance

**Figure 5:**
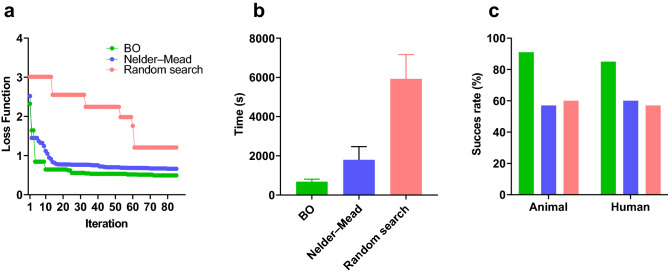
Performance comparison in three optimisation methods: Bayesian optimisation (BO), Nelder–Mead and random search. Panel (**a**) shows the loss function of three methods analysed in one data set. Panel (**b**) shows the mean and variance of the time consumptions of three techniques in three random data. Panel (**c**) shows the success rate (the percentage of optimisation solutions in total) of each method in three data sets.

Figure 5 compares the efficiency and time consumption among optimisation methods (Bayesian optimisation, Neider–Mead and random search). Panel a shows one example of the loss function versus iterations in one example data set. The Bayesian optimisation can provide more accurate approximations in less than 15 min compared to others. In panel c, the success rate is the percentage of the meaningful results in total. This rate was higher in Bayesian optimisation when compared to other methods (95% in animal data and 82% in human set).

## Discussion

Overall, this study has quantified lung heterogeneity using a non-invasive lung function test, the Inspired Sinewave Test, combined with advanced computational methods. Heterogeneity values were captured and demonstrated in both animal and human studies, in both health and disease. In this section, we discuss in detail the method to calculate the lung heterogeneity noninvasively, and consider the use of these indices as a tool to classify lung conditions.

Ventilatory heterogeneity is the mismatch in distribution of the ventilation relative to compartment size. The latter is sometimes referred to as ‘specific ventilation’ and represents the reciprocal of the time-constant of a compartment. Perfusion heterogeneity is mismatch of perfusion relative to compartment size. Both of these forms of heterogeneity are related to, but distinct from, V/Q heterogeneity which is the non-uniform distribution of V/Q ratios (with respect to perfusion or ventilation), which is not the subject of this paper. In this work, the standard deviation of the lognormal distributions of ventilation and perfusion (${\sigma }_{V}$ and ${\sigma }_{P}$) was shown to adequately represent and usefully quantify heterogeneity of both ventilation and perfusion (both relative to compartment size). Such heterogeneity has previously been shown to be represented by a lognormal distribution. An example of this is the Multiple breath Nitrogen Washout technique; a relatively technically complex technique which assumes this distribution^11,24^. Ventilatory heterogeneity could be usefully presented to clinicians in both numerical format (standard deviation) and visually, in the form of a lognormal plot. The lognormal distribution is straightforward to understand and easily recognised by clinicians. Thirdly, this distribution could be upgraded easily in the future to allow for bi-modal or multimodal distributions.

Our results for the ventilation and perfusion lognormal distributions agreed with previous studies. Other research using different lung measurement technique has shown similar standard deviations of ventilation and perfusion of $0.53$ and $0.55$ in healthy people, albeit in a much smaller sample ($n=6$)^9^. In our study, in Fig. 3, healthy people had mean values of ${\mathrm{\sigma }}_{v}=0.71$ and ${\mathrm{\sigma }}_{p}=1.0$. Because age is an important factor in COPD, in Fig. 3c, we were able to compensate for this effect in future analyses. The outlying results in the confusion matrix for the GOLD phenotypes were never greater than $\pm 1$, indicating generally good agreement. Heterogeneity indices (expressed as % predicted for age) were significantly different between healthy and COPD cohorts. Overall, % predicted $\sigma _{v}  = 100~ \pm 17$ (healthy) versus $\sigma _{v}  = ~140 \pm 26$ (pooled COPD).

In the human data set (classified according to ‘GOLD’), the result was not accurately classified by the IST heterogeneity indices. This is likely because COPD represents a wide variety of pathology and severity, and further still, the GOLD classification is based on spirometry and reported symptoms which themselves have an unknown relationship to pathological severity. Age is known to be an essential factor in COPD disease prediction^19^. Our research shows similar results. We have also demonstrated a correlation between specific ventilatory heterogeneity and perfusion heterogeneity versus age in healthy participants. Thus, although we normalised the lung heterogeneity indices to account for the effect of age, genetic factors, tobacco smoke and outdoor air pollutants play essential roles in the patients’ lung heterogeneity^25^, which could not be controlled for. In contrast, however, there was greater uniformity in experimental conditions both before and after lung injury, which would have allowed the specific effects of injury to be more evident in the animal experiments.

Although the optimisation process was time-consuming, Bayesian optimisation boosted the performance of the lung simulation-based optimisation model. A similar optimisation problem has been tackled before, but the time and computing power required were prohibitive^9^. However, we have demonstrated that Bayesian optimisation could calculate results in ten minutes using a standard office computer. In addition, our approach was more effective in dealing with the potentially non-convex optimisation problem than the Nelder-Mead. Our advanced optimisation method not only improves the diagnostic ability of the IST but also its translation to human medicine.

In this work, we demonstrated the ability of the IST, a relatively simple to implement lung function test, as a diagnostic tool. The IST results are reliable and clinically meaningful^17,18^. Previous studies indicated that, in ventilated subjects, the IST could measure the effective lung volume (“baby lung”) and capture the pulmonary blood flow trend^17,18^. The IST does not require special, forced manoeuvres, which patients often find challenging and uncomfortable. In an assessment of comfort (n = 18), 78% of participants selected the IST as their preferred test comparing to spirometry and body plethysmography^26^. The ability of IST to measure ventilatory heterogeneity could improve the impact of the IST when used as a diagnostic tool. For example, in Fig. 4c, a simple multi-class logistic classification was implemented. If we assumed that the GOLD classification (based on the spirometry values) was a ground truth^6^, the classification result using the IST heterogeneity values could predict accurately 100% of healthy patients and 73% overall.

Furthermore, the IST also could classify ARDS lungs (injured) versus control (noninjured), the $AuC = 0.76~(p < 0.0001)$ of the ROC curve (in Fig. 3d). Application of IST could benefit patients undergoing mechanical ventilation. Clinicians could use IST measured values to optimise ventilator settings to match the individual patient’s lung conditions.

This study has several strengths. We included a large number of data sets from IST (13 animals and 104 participants). This work is the first study to provide detailed lung heterogeneity data in ARDS and COPD patients using the IST. However, we acknowledge several limitations. The included data were collected during experiments designed for other purposes. Moreover, because the IST is a gas exchange technique, and N_2_O has only modest blood solubility, the influence of perfusion is weaker than that of ventilation. This is likely to yield less reliable parameter recovery values than ventilation data (results presented in Fig. 2b, d). Additionally, our model results contained two sets of parameters: the parameters to control the shape of the lognormal distributions and the absolute physiological lung parameters. Because this paper aims to report the heterogeneity of the lung, the volumetric lung parameters will be reported in another study. Furthermore, our lung simulation and its lognormal assumptions might not perfectly fit the measured data. This lognormal distribution was assumed to be an accurate representation of the lung heterogeneity^27,28^. But, in several patients, the distribution of lung heterogeneity was recorded in the bimodal log-distribution. Therefore, future work will aim to loosen the prior assumptions of the distribution and to accommodate alternative possibilities. Future comparison of IST ventilation heterogeneity and perfusion heterogeneity indices with those measured by CT scanning will be performed^29,30^. Sophisticated lung simulations or advanced optimisation methods could also be introduced in the future. However, the required time and computer power consumption might then be considerable.

We have presented a new approach to quantify ventilatory heterogeneity using Bayesian optimisation combined with a non-invasive lung function test (the Inspired Sinewave Technique). We have shown that the IST can measure heterogeneity indices and we applied this model to further explore lung heterogeneity in health and disease. Our results support further development of this technique and its translation to human medicine. The need to measure the lung volume of mechanically ventilated patients and to personalise ventilation, may aid in reducing driving pressure and therefore mortality in patients with COPD and acute respiratory distress syndrome (ARDS) in the future.

## Methods

A lung simulation was developed to work with the Inspired Sinewave Technique. The IST compares the tracer gas (N_2_O) inhaled and exhaled signals and applies an analytical model to calculate cardiopulmonary parameters^18,31,32^. In theory, the analytical model should be invertible, and we should be able to recalculate the lung parameters from this model. However, in practice, the complexity of signal processing, the signal to noise ratio and the non-convexity of the optimisation means that inversion of the model is not practicable. Therefore, we developed a simulation-based optimisation and applied Bayesian optimisation to quantify the ventilation and perfusion heterogeneity using the IST results.

The Bayesian optimisation was then applied to match simulated to measured data so creating a simulation-based optimisation model. The model was then verified by simulated patient data from the literature (emphysema and pulmonary embolism). A simulated emphysema patient’s lung displayed abnormal ventilation heterogeneity, and similarly, a simulated pulmonary embolism patient revealed abnormal perfusion heterogeneity^33^. A schematic diagram of the model and results of simulated heterogeneous lungs are shown in Fig. 1.

When the model was completed and verified, we applied this to calculate lung heterogeneity indices from our IST patient and animal studies data. The characteristic summary of the animal and human data is presented in Table 2. We executed the IST measurements at two tracer gas oscillation frequencies (periods 180s and 60s). All IST measurements and analysis were carried out in accordance with relevant guidelines and regulations in the study.

**Table 2:** Characteristic summary of the animal and human data measured by the IST.

Parameter	Noninjured	Injured
**Animal**		
Number	13	13
Weight (kg)	29 (2)	–
Heart rate (bpm)	86 (12)	85 (11)
pH	7.42 (0.03)	7.28 (0.09)
F_I_O_2_	0.4 (0.1)	0.8 (0.1)
Cardiac output (L/min)	3.4 (0.8)	3.2 (0.4)

	Healthy	COPD
**Human**		
Number	32	72
Age	53 (22)	67 (10)
Height (m)	1.7 (0.1)	1.7 (0.1)
Weight (kg)	72 (12)	79 (18)
BMI	25 (4)	28 (5)

Mean (SD) are shown. F_I_O_2_ is the fraction of inspired oxygen.

Animal data were obtained from 13 anaesthetised pigs which were studied by the IST both before lung injury (noninjured) and after lung injury (injured). Experiments were performed at the Hedenstierna Laboratory, Uppsala University, Uppsala, Sweden. It was approved by the regional animal welfare and ethics committee (Ref: C98/16). Lachmann’s method was applied to induce lung injury to model the ARDS^34^. Details of the animal preparation can be found in^18^. PEEP was applied at 5, 10, 15 and 20 cmH_2_O incrementally. The fraction of inspired oxygen (FiO_2_) was kept unchanged at 0.4 before injury and 0.8 after injury to maintain oxygenation. ﻿Reporting in this paper adheres to the Animal Research: Reporting of In Vivo Experiments guidelines^35^.

The human clinical data used here comprised two datasets taken from another research study^19,36^. Informed consent was obtained from all subjects. We analysed data from 104 participants (32 Healthy and 72 COPD). COPD patients met the inclusion criteria if they were aged > 40 years, had a 10-pack year smoking history, and a FEV_1_/FVC < 0.7. Patients were classified into the four “GOLD” categories (1–4) based upon FEV1%^2,6,37^. The protocol for the study was approved by an NHS ethical committee (16/SC/0057, South Central—Hampshire B Research Ethics Committee) and conformed to the Declaration of Helsinki, 2013. All procedures for each participant were performed on the same day within the Respiratory Medicine Department of the Churchill Hospital, Oxford University Hospitals.

Finally, to compare the optimisation performance, three optimisation methods (Bayesian optimisation, Nelder-Mead and random search) were performed using the same desktop computer (Intel core I7, 32 GB of RAM).

### The lung simulation-based optimisation model

Figure 1a presents a schematic diagram of the lung simulation-based optimisation model. This model contains two parts, which are the lung simulation and the optimisation. The lung was simulated by ten dead-space compartments, 125 alveolar compartments (tidally ventilated) and five body compartments. A simple version of this lung simulation was implemented and validated previously in^31,38^. In this work, we increased the number of alveolar compartments to 125 and applied the fraction of ventilation and perfusion to each individual compartment to represent the heterogeneity of the lung. These fractions were described and generated by a lognormal distribution^9^.

Some of the parameters of this lung simulation were taken directly from the patient's record, such as tidal volume, respiration rate, inspiration-expiration ratio and deadspace volume (measured by the IST). The unknown parameters are alveolar volume, pulmonary blood flow and the width (i.e. the log Standard Deviation) of the ventilation and perfusion heterogeneity lognormal distribution, which were the focus in the optimisation.

The concept of the lognormal lung has been presented elsewhere^9^. In this paper, we introduce a simple lognormal distribution of the lung to show lung heterogeneity. For example, in Fig. 1b, in a healthy patient, the log standard deviation of the specific ventilation heterogeneity (${\sigma }_{V}$) was found to be 0.3 and the log standard deviation of the perfusion heterogeneity (${\sigma }_{P}$) was found to be 0.5^9^. In this lognormal distribution, the more damaged the lung, the wider the distribution (larger ${\sigma }_{V}$ or $\mathrm{\mu }$ being further away from 1). The shape of the distribution depends on $\mathrm{\sigma }$ and $\mathrm{\mu }$.

We used Bayesian optimisation to match the data from the simulated lung to that of the measured data to calculate these unknown parameters^39,40^. A loss function showed the difference between the simulated and measured values. Thus, the Bayesian optimisation can statistically suggest parameters for the simulation to minimise the loss function. Minimising the loss function $f\left(\mathrm{\theta }\right)$ can estimate the input parameters $\mathrm{\theta }$, including $\left({V}_{A},{Q}_{P},{\mathrm{\sigma }}_{V},{\mathrm{\mu }}_{V},{\mathrm{\sigma }}_{P},{\mathrm{\mu }}_{P}\right)$, where ${V}_{A}$ is alveolar volume and ${Q}_{P}$ is pulmonary blood flow,

3 $$arg\left(\left|\frac{{ELV}_{180}^{Sim}\left(\theta \right)-{ELV}_{180}^{M}}{{ELV}_{180}^{M}}\right|+\left|\frac{{ELV}_{60}^{Sim}\left(\theta \right)-{ELV}_{60}^{M}}{{ELV}_{60}^{M}}\right|+\left|\frac{{Qp}_{180}^{Sim}\left(\theta \right)-{Qp}_{180}^{M}}{{Qp}_{180}^{M}}\right|+\left|\frac{{Qp}_{60}^{Sim}\left(\theta \right)-{Qp}_{60}^{M}}{{Qp}_{60}^{M}}\right|\right)$$

where $EL{V}_{180}^{Sim}\left(\mathrm{\theta }\right)$ is the simulated effective lung volume value based on the set parameter $\mathrm{\theta }$ and $EL{V}_{180}^{M}$ is the measured effective lung volume from the patient. The $EL{V}_{180}^{Sim}\left(\mathrm{\theta }\right)$ is the result of the IST running on a simulation lung with known conditions (parameter θ). Similarly, with ${Q}_{P,180}^{Sim}\left(\mathrm{\theta }\right)$ and ${Q}_{P,180}^{M}$. 180s and 60s were the periods of the IST tracer gas oscillation. Further details of the lung model development and the Bayesian optimisation are included in the appendix.

### Statistical analysis

Statistical analyses were conducted using GraphPad Prism (GraphPad Software inc.) and Matlab v2018b (Mathworks, Natick, MA, USA). To compare differences between healthy participants and patients in the 1–4 GOLD classifications, Kruskal–Wallis test was used^41^. To compare two groups, we used an unpaired *t *test. The Area under The Curve (AuC) of the Receiver Operating Characteristics (ROC) curve was used to measure the ability of our test to distinguish between healthy and COPD^42^. Furthermore, we also applied a maximum likelihood estimation of the logistic regression model using only our IST outputs to classify participants as healthy or into the 1–4 GOLD categories^43,44^.

## Data availability

Data available upon request.

## Supplementary Information

Supplementary Information.
