# Supplementary material for: Simulation-based optimisation to quantify heterogeneity of specific ventilation and perfusion in the lung by the Inspired Sinewave Test
Source: Sci Rep. 2021 Jun 16;11:12627. doi: 10.1038/s41598-021-92062-w (PMC8208972; doi:10.1038/s41598-021-92062-w)
Supplement: Supplementary file 1 — Supplementary Information. [file 41598_2021_92062_MOESM1_ESM.pdf]

Supplementary information

A1. Lung simulation

Overall, this lung simulation requires an input, which is the N_2O concentration of IST inspired tracer gas, $F_I(t)$, and generates an output: the expired gas concentration signal, $F_E(t)$. Figure A1 shows a schematic diagram of the IST and the lung simulation. This lung simulation contains three main compartments: deadspace, alveolar and body compartments. Harrison showed that the mixing model of the deadspace with two compartments provided better results [1]. The same study has showed that there is no significant change when the number of deadspace compartments is more than ten. Therefore, ten mixing compartments of deadspace are included in this model. The *ode45* solver and Simulink-Matlab were chosen for simulation.

The whole deadspace, alveolar and body compartments are linked together by governing equations which represent the equilibrium of the mass concentration of the tracer gas. $C_{\bar{v}}$ and C_a are the mixed venous and pulmonary end-capillary gas concentrations, respectively. Inspiration (A1) and expiration (A2) are:

$$\frac{d}{dt}\big(V_A(t) \times F_A(t)\big) = f_{ven,i} \times \dot{V_{A,\iota}}(t) \times F_{IA}(t) + f_{per,i} \times \dot{Q_{p,\iota}}(C_{\bar{v}} - C_a) \tag{A1}$$

$$\frac{d}{dt}\big(V_A(t) \times F_A(t)\big) = f_{ven,i} \times \dot{V_{A,\iota}}(t) \times F_A(t) + f_{per,i} \times \dot{Q_{p,\iota}}(C_{\bar{v}} - C_a) \tag{A2}$$

where i is the number of tidal lung compartments, each compartment has their own fraction of ventilation $f_{ven,i}$ and fraction of perfusion $f_{per,i}$. $F_{IA}(t)$ is the fraction concentration coming to the lung compartment (from deadspace compartment). $F_A(t)$ is the fraction of concentration coming back from to alveolar compartment to the deadspace compartment during expiration.

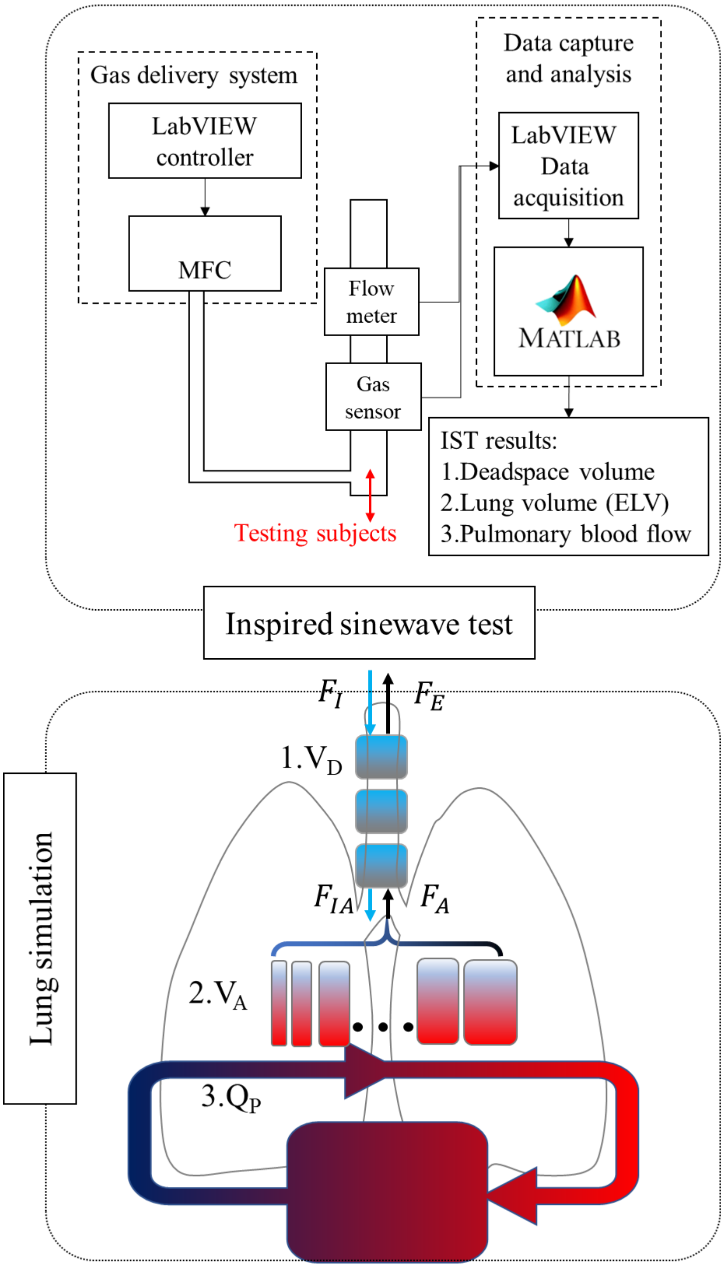

Figure A1: A schematic diagram of the Inspired Sinewave Test and Lung simulation. In the lung simulation, V_D is simulated by three deadspace volume compartments. V_A is simulated by 125 alveolar compartments. Q_P is the pulmonary blood flow and the connection to the body compartment.

These fractions for ventilation and perfusion are calculated from the log-normal distribution [2].

$$f(x) = \frac{1}{x} \times \frac{1}{\sigma\sqrt{2\pi}} exp\left(-\frac{(ln\, x - \mu)^2}{2\sigma^2}\right) \quad \text{(A3)}$$

where $f(x)$ is the ventilation or perfusion fraction of the compartment x. Parameter σ and μ could be defined by the mean m and variance v of the lognormal distribution:

$$\mu = log\left(\frac{m^2}{\sqrt{v + m^2}}\right) \quad \text{(A4)}$$

$$\sigma = \sqrt{log\left(\frac{v}{m^2 + 1}\right)} \quad \text{(A5)}$$

Inspired sinewave technique operates by delivering a precise bolus dose of N_2O via a mass flow controller to the lung via the inspired breath and analysing the acquired output [3]. The testing subject inhales and exhales air through a mainstream tube, which is equipped with a flow meter and a gas sensor. IST analysis was mentioned elsewhere [4]. The tracer gas was delivered in the form of sinewave equation:

$$F_I(t) = IST_A + IST_B \times sin\left(\frac{2\pi \times t}{IST_T}\right) \quad \text{(A6)}$$

where IST_A is the average of the N_2O tracer gas, IST_B is the amplitude of the tracer gas and IST_T is the time period of the sinewave tracer gas.

A2. Bayesian Optimisation

Bayesian optimisation views an experiment as a black-box function $y = f(x)$ which takes an input x (design choice) and produce an outcome y (target measurement) to be optimised (Figure A2). Fundamentally, Bayesian optimisation is a sequential model-based approach to optimise the black-box function. Bayesian optimisation prescribes a prior belief over the

possible objective functions using a Gaussian process regression model [5] and then sequentially refines this model as data are observed via Bayesian posterior updating. Equipped with this probabilistic model, we can sequentially guide exploration using a decision function that leverages the uncertainty in the posterior.

Formally, let $f: \mathcal{X} \rightarrow \mathcal{R}$ be a well-behaved function defined on a subset $\mathcal{X} \subseteq \mathcal{R}^d$. Our goal is to solve the following global optimisation problem $x^* = \arg\max_{x \in \mathcal{X}} f(x)$. As a black-box function, we do not have a closed-form expression for f and it is computationally expensive to evaluate. We summarise the algorithm and illustration for BO in Figure A2.

Gaussian process

Bayesian optimisation reasons about f by building a Gaussian process (GP) through evaluations [5]. This flexible distribution allows us to associate a normally distributed random variable at every point in the continuous input space. We get the predictive distribution for a new observation x' that also follows a Gaussian distribution where we can estimate the predictive mean $\mu(x)$ indicating the expected function value and predictive variance $\sigma(x)$ indicating the uncertainty

Algorithm 1 Bayesian Optimisation

initial data $\mathcal{D}_0$, #iter T

for $t = 1$ to T **do**
  Fit a surrogate model as a GP from $\mathcal{D}_t$
  Build an acquisition function from this GP
  Select a next query $\mathbf{x}_t = \arg\max \alpha(\mathbf{x})$
  Evaluate $y_t = f(\mathbf{x}_t)$
  Augment $\mathcal{D}_t = \mathcal{D}_{t-1} \cup (\mathbf{x}_t, y_t)$
end for

Output: $\mathbf{x}_{\max}, y_{\max}$

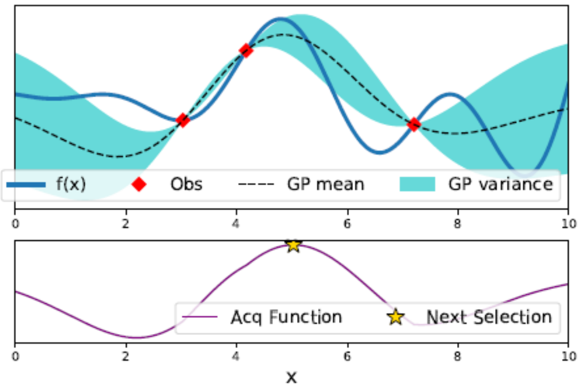

Figure A2: Bayesian optimisation coding example and graph. Left: Algorithm for Bayesian optimisation using Gaussian process (GP) as a surrogate model to make a query for evaluation. Right: Illustration of BO given three initial observations in red.

Acquisition functions

We then construct the acquisition function $\alpha(x)$, from the GP surrogate model above, to determine a next point to evaluate. The acquisition functions are carefully designed to optimise the trade-off between expanding the search space, and exploring *current* promising regions. A simple example of the acquisition function is the upper confidence bound (UCB) $\alpha(x) = \mu(x) + \kappa\sigma(x)$ where κ is the parameter controlling the exploration. Another common acquisition function is the expected improvement [6,7] where we consider the expectation of improving over a certain threshold y^+ as $E[\max(0, f(x) - y^+)]$. Using the analytical form of the Gaussian distribution, we can derive the closed-form computation of the EI, as shown in [8].

We then maximise the acquisition function to select the next point $x_{t+1} = \arg\max_{x \in \mathcal{X}} \alpha_t(x)$. In this auxiliary maximisation problem, the acquisition function form is known and can be easily optimized by standard numerical techniques.

A3. Verification of the lung simulation

In Figure A3, the verification results of the model show that this model can work accurately with the IST. The linear regression analysis of the model outputs with the simulated true inputs indicated strong agreement and high correlation values. In panels a and b, according to simulated data results, our model can calculate values for alveolar volume and pulmonary blood flow ($R^2 = 0.99$). Furthermore, in the results for the specific ventilation heterogeneity and perfusion heterogeneity or the shape of the lognormal distribution in panels c and d, good agreement was found between model input and output (σ_V has $R^2 = 0.7$ and σ_P has $R^2 = 0.9$). Therefore, this model can measure the specific ventilation heterogeneity and perfusion heterogeneity in simulated data and present it in the form of σ_V and σ_P. These values can be used to form the lognormal distribution for visual presentation.

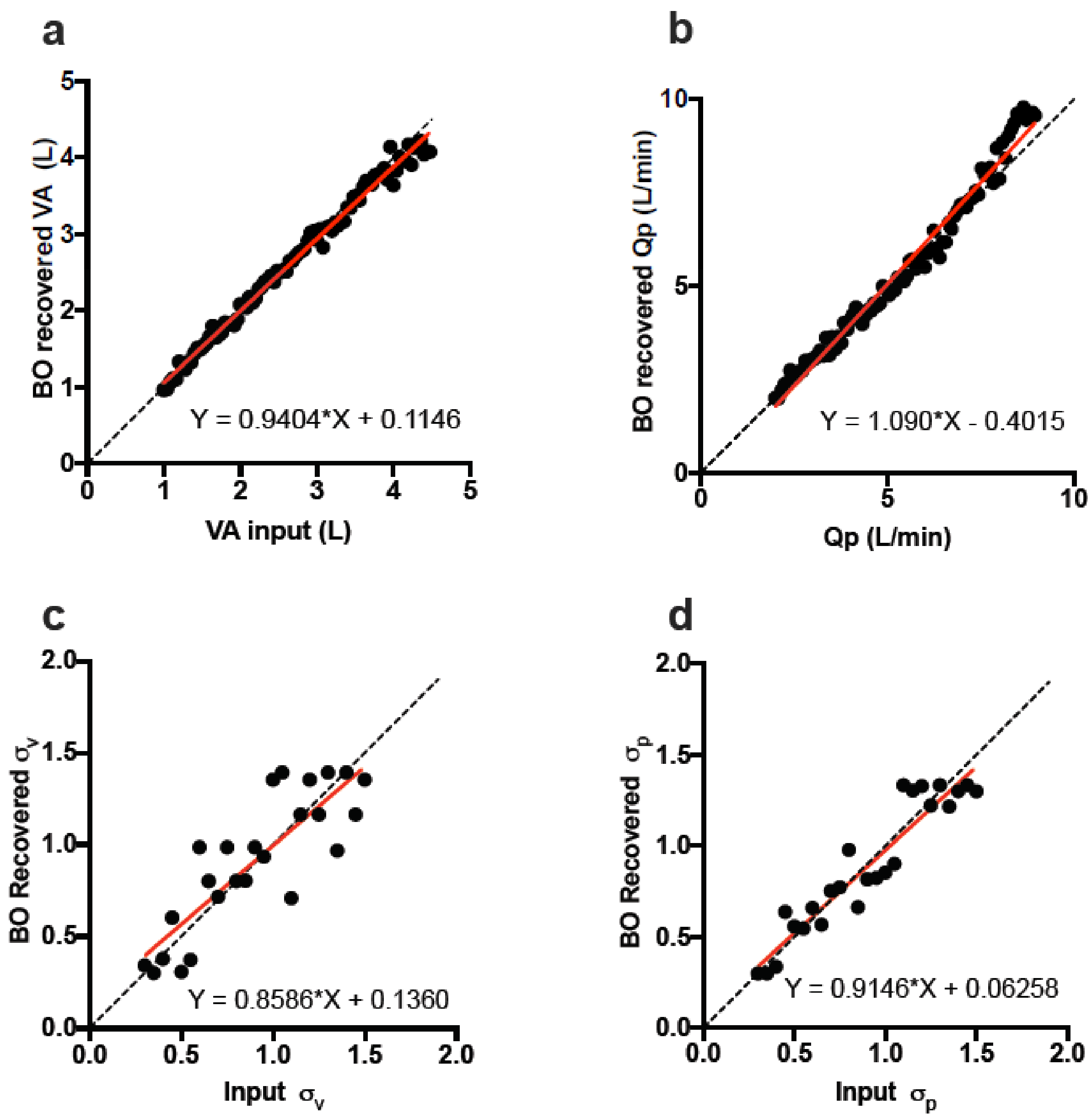

Figure A3: Verification of the lung simulation. Linear regressions present the relationship between set-input and recovered output calculated by the Bayesian optimisation lung simulation. Black dash lines are reference lines and red lines are regression lines. The simulation contains Gaussian random noise in the tidal volume to represent the real patient breathing pattern.

References

1. Harrison, C. D. *et al.* Modelling mixing within the dead space of the lung improves predictions of functional residual capacity. *Respir. Physiol. Neurobiol.* **242**, 12–18 (2017).

2. Mountain, J. E. *et al.* Potential for noninvasive assessment of lung inhomogeneity using highly precise, highly time-resolved measurements of gas exchange. *J. Appl. Physiol.* **124**, 615–631 (2018).

3. Phan, P. A. *et al.* The Inspired Sinewave Technique: A Comparison Study With Body Plethysmography In Healthy Volunteers. *IEEE J. Transl. Eng. Heal. Med.* **5**, (2017).

4. Bruce, R. M. *et al.* Noninvasive cardiac output monitoring in a porcine model using the inspired sinewave technique: a proof-of-concept study. *Br. J. Anaesth.* **123**, 126–134 (2019).

5. Rasmussen, C. E. Gaussian processes for machine learning. (2006).

6. Jones, D. R., Schonlau, M. & Welch, W. J. Efficient global optimization of expensive black-box functions. *J. Glob. Optim.* **13**, 455–492 (1998).

7. Bull, A. D. Convergence rates of efficient global optimization algorithms. *J. Mach. Learn. Res.* **12**, 2879–2904 (2011).

8. Nguyen, V., Gupta, S., Rana, S., Li, C. & Venkatesh, S. Regret for Expected Improvement over the Best-Observed Value and Stopping Condition. in *Proceedings of The 9th Asian Conference on Machine Learning (ACML)* 279–294 (2017).
